# Indirubin combined with umbilical cord mesenchymal stem cells to relieve psoriasis-like skin lesions in BALB/c mice

**DOI:** 10.3389/fimmu.2022.1033498

**Published:** 2022-11-16

**Authors:** XiaoJuan Lu, Hao Wang, Hongwei Wang, Fan Xie, Cuibao Jiang, Danpeng Shen, Hongpeng Zhang, Jie Yang, Youshu Lin

**Affiliations:** ^1^ Department of Dermatology, Guangzhou Red Cross Hospital of Jinan University, Guangzhou, China; ^2^ Lab Animal Research Center, Asia Stem Cell Regenerative Pharmaceutical Co. Ltd., Shanghai, China; ^3^ Department of Dermatology, The Fifth People’s Hospital of Hainan Province, Haikou, China

**Keywords:** psoriasis, indirubin, umbilical cord mesenchymal stem cells, combined administration, inflammatory factor

## Abstract

**Objective:**

To investigate the efficacy of indirubin combined with human umbilical cord mesenchymal stem cells (hUC-MSCs) in the treatment of psoriatic lesions in BALB/c mice and to explore the related mechanism of indirubin in the treatment of psoriasis.

**Methods:**

A BALB/c mouse psoriasis model induced by imiquimod was established and randomly divided into the control group, model group, indirubin group, hUC-MSCs group, and indirubin combined with hUC-MSCs group. Psoriasis area and severity index (PASI) score was used to observe skin lesion changes in the psoriasis-like mouse model. The epidermal scale, the degree of keratinization, and the infiltration of inflammatory cells were observed by hematoxylin eosin (HE) staining. The concentrations of TNF-α, IFN-γ, IL-17A, and IL-23 in serum of mice were measured using enzyme-linked immunosorbent assay (ELISA).

**Results:**

The PASI integral trend chart indicates that hUC-MSCs and indirubin and the combination of drugs could relieve the appearance of skin lesions and accelerate the recovery of skin lesions. The indirubin group had the best effect in improving the scale of skin lesions. HE staining showed that the number of parakeratosis cells in the three treatment groups was significantly reduced, the degree of erythrocyte extravasation dermis hyperplasia and inflammatory cell infiltration was significantly lower than that in the model group, and the skin thickness and spleen index of the combined treatment group exhibited the most noticeable improvement. ELISA showed that the concentrations of TNF-α, IFN-γ, IL-17A, and IL-23 in serum of mice in the hUC-MSCs treatment group, indirubin group, and combined administration group were all decreased compared with those in the model group, and the concentrations of IFN-γ, IL-17A, and IL-23 could be decreased significantly in the indirubin group.

**Conclusions:**

Both hUC-MSCs and indirubin can effectively reduce psoriasis-like lesions in BALB/c mice, and the combined administration of these drugs has the best effect.

## 1 Introduction

Psoriasis is an immune-mediated chronic, recurrent, inflammatory skin disease. It’s typical clinical manifestations are scaly erythema or plaques, which may be localized or widely distributed. The etiology of psoriasis involves various factors such as heredity, immunity, and environment. Excessive proliferation of keratinocytes or inflammation of synovial cells and chondrocytes in joints is caused by the immune response mediated mainly by T lymphocytes and jointly participated by various immune cells. Psoriasis is an incurable disease, and many therapeutic drugs and methods are available at present. The treatment plan of psoriasis should be determined according to the symptoms of patients; mild patients can undergo external treatment, moderate and severe patients can use system treatment, and patients that respond poorly to traditional systemic drug treatment can choose targeted biological treatment. The selection of drugs and methods suitable for patients is important to control the disease and maintain long-term efficacy (1).

Although great progress has been made in the study of the pathogenesis of psoriasis in recent years, there is still a long way to go before the pathogenesis of psoriasis is fully elucidated. The high responsiveness of Th1 and Th17, the dysregulation of Tregs, and the complex relationship among immune system cells, keratinocytes, and vascular endothelial cells play an important role in the pathogenesis of psoriasis (2). The interleukin-23/Th17/IL-17 axis and Th1/IFN-γ axis play a key role in the inflammation of psoriasis. A series of biologics has been developed and put into use for the above immune mechanisms. However, the current biologics targeted therapy cannot completely cure psoriasis, which manifests in a small number of patients who are not sensitive to drugs or gradually develop treatment resistance. Therefore, the treatment of psoriasis is still under constant exploration (2).

The role of stem cells has attracted increasing attention. The effects of epidermal stem cells and stem cells on T cells have been found. The dysfunction of various types of stem cells may be the main reason for the dysregulation of inflammatory response in psoriasis (3, 4). Human umbilical cord mesenchymal stem cells (hUC-MSCs) are characterized by low immunogenicity, high expansion rate *in vitro*, and pluripotent differentiation potential, and they can be used as the ideal choice of mesenchymal stem cells for the treatment of psoriasis. Previous studies found that hUC-MSCs can produce IL-35 and induce the proliferation of Treg cells, thereby reducing the active expression of Th17 and Th1 cells (5, 6). Study also found that subcutaneous injection of hUC-MSCs may reduce the production of IL-17 by inhibiting γδT cells, thereby inhibiting skin inflammation and having therapeutic effects on psoriasis (7).

Due to the increasing application of Traditional Chinese Medicine in diseases, a variety of Traditional Chinese Medicine ingredients have been proved to have therapeutic effects on psoriasis, such as total glucosides of paeony, coptis coptidis, curcumin and so on (8, 9). Indigo naturalis has the functions of heat clearing, detoxifying, cooling blood, and calming nerves, and it is widely used in modern medicine to treat inflammation-related and autoimmune diseases. The external application of indigo naturalis ointment can significantly improve the erythema, scale, and infiltration of skin lesions in patients with psoriasis vulgaris and significantly reduce the psoriasis area and severity index (PASI) score. Its efficacy is proportional to the use time and concentration of indigo naturalis, and its safety is good (10, 11). Qingdai oil can improve psoriasis damage to nails (12, 13). Indirubin is a bisindole compound extracted from the leaves of indigo, which together with indigo constitutes the main active component of indigo naturalis. Many studies have shown that indirubin can inhibit inflammatory response and reduce psoriasis-like skin injury in mice (14, 15). Indirubin is stable and effective in the treatment of psoriasis, and can be used as a new topical agent for the treatment of psoriasis.

By observing the therapeutic effect of indirubin combined with stem cells in the treatment of psoriasis-like skin lesions in BALB/C mice, this paper aims to elucidate the immune mechanism of indirubin combined with stem cells in the treatment of psoriasis and the mechanism of regulating psoriasis-like inflammation.

## 2 Experimental materials and methods

### 2.1 Primary reagent

For the experiments, 5% imiquimod cream (Batch No. 17010139) was purchased from Sichuan Mingxin Lidi Co., LTD. Recombinant mouse interleukin-12 (RMIL-12 p70) was purchased from BD Pharmingen Inc (Batch No. 6278771, specification: 5 μg). Lipopolysaccharide (LPS) lyophilized powder was purchased from Sigma Company, USA (Batch No. 045M4087V; specification: 10 mg). IL-17A, IL-12 enzyme-linked immunosorbent assay (ELISA) kits were purchased from Shenzhen Dakewe Bioengineering Co., LTD (Batch No. DKW12-2170-048, DKW12-2020-048). Indirubin (Batch No. 110717-201805) was purchased from China Institute for Food and Drug Control and diluted at 0.5% CMC for use. Multiple Electrolytes Injection was purchased from Shanghai Kelun Pharmaceutical Co., Ltd. (Batch No. T14102120).

HUC-MSCs were produced by the GMP laboratory of Shanghai Quansheng Biotechnology Co., Ltd. The umbilical cord donor had signed an informed consent form for donation for scientific research and passed infectious disease tests. The collected umbilical cord tissue was cut and separated by cell climbing. After passage culture and expansion, 1 × 10^8^ cells/mL saline cell suspension was prepared (Batch No. 20191201). The extracted hUC-MSCs were cultured in DMEM medium supplemented with 10% fetal bovine serum and 100 U/ml penicillin/streptomycin. After passing the quality inspection of sterility, mycoplasma, endotoxin, and molecular phenotyping, it was used in this experiment. The experimental instruments were provided by Shanghai Quansheng Biotechnology Co., Ltd.

### 2.2 Experiment animals

Fifty SPF male BALB/c mice aged 6–8 weeks and weighing about 20 g were purchased from Beijing Weitong Lihua Laboratory Animal Co., Ltd. Experimental animals were kept in the laboratory for at least 1 week and then tested under standard feeding conditions (room temperature 25°C–27°C) with alternating 12 h light and dark cycles. Free access to food (standard pellet feed) and water was allowed. Experiments were performed under a project license (No. 2016LL003) granted by institutional board of The Fifth People’s Hospital of Hainan Province, in compliance with The Fifth People’s Hospital of Hainan Province guidelines for the care and use of animals.

### 2.3 Animal model establishment and grouping

Animal protection and use guidelines were followed during the experiment. Fifty SPF male BALB/c mice were randomly divided into five groups: group A (blank group), group B (model group), group C (hUC-MSCs group), group D (indirubin group), and group E (indirubin + hUC-MSCs group), with 10 animals in each group. In group B, 5% imiquimod cream was applied to the back of the mice at 62.5 mg/day for 7 days. At the beginning of modeling, RMIL-12 10 ng (5 μL) and LPS 20 μg (5 μL) were injected subcutaneously once to make the psoriasis-like rash of mice more durable and typical (16). On the basis of the same modeling as group B, 2 × 10^7^/kg hUC-MSCs were injected through the tail vein in group C on the first day. Group D was given the indirubin solution (50 mg/kg, 200 μL, 0.5% CMC dilution) by gavage once a day. In group E, stem cell preparation was injected once through the tail vein on day 1, and indirubin solution (50 mg/kg, 200 μL, 0.5% CMC dilution) was administered by gavage once a day. All mice were sacrificed after observation on day 7, and the blood and skin tissue were collected for use. The spleen was taken and weighed, and the spleen index (spleen mass/body mass) was calculated.

### 2.4 Stemness identification of hUC-MSCs

Fluid identification: The second passage cells were collected and digested with 0.25% TrypLE trypsin (Gibco), and the appropriate amount of cells was incubated with CD73, CD90, CD105, CD34, CD45, and HLA-DR antibodies (1 μg/100 μL, CST) conjugated to PE luminescent groups for 30 min on ice. Flow cytometry (Beckmancoulter) was used to detect the level of antibodies on the cell surface.

Osteogenic induction: hUC-MSCs were seeded in a six-well plate at a density of 1 × 10^5^ cells/well. In addition, 10 mmol/L β-sodium glycerophosphate, 1 μmol/L dexamethasone, and 50 μmol/L vitamin C were added to the conventional medium as the osteogenic induction medium. Cells were cultured in osteogenic induction medium for 4 weeks, and mineral bone nodules were detected using Alizarin Red S dye.

Adipogenic induce supplements: hUC-MSCs were seeded in a six-well plate at a density of 1 × 10^5^ cells/well. The conventional medium was supplemented with 0.5 mmol/L isobutyl methyl xanthine, 200 μmol/L indomethacin, 1 μmol/L dexamethasone, and 10 μmol/L insulin as adipogenic induction medium. After the cells were cultured in adipogenic induction medium for 1 week, lipid droplets were detected using oil Red O dye.

Chondrogenic induction: hUC-MSCs cells were seeded in a six-well plate at a density of 1 × 10^5^ cells/well. The conventional medium was supplemented with 10 μg/L transforming growth factor-β1, 0.1 μmol/L dexamethasone, 100 μmol/L ascorbic acid, 200 mmol/L glutamine, and 1% ITS as chondrogenic induction medium.

### 2.5 Appearance scoring detection

The skin lesions of mice in each group were observed with naked eye and photographed with camera every day before drug administration. PASI was used to evaluate the appearance of mice, including three subscales of erythema, scale and skin hypertrophy. The total PASI score was obtained by adding the scores of the three subscales. At the same time, two professional technicians besides the experimenter were asked to evaluate the degree of skin injury of the mice in a blind way. The scoring criteria are shown in **Table 1**.

**Table 1:** PASI scoring criteria.

Symptom	Standard for evaluation
Erythema	0 points: No erythema
	1 points: Blush
	2 points: Red
	3 points: Deep red
	4 points: Very dark red
Skin hypertrophy	0 points: The lesions are level with normal skin.
	1 points: The skin lesions are slightly higher than the normal skin surface.
	2 points: The skin lesions have a medium elevation.
	3 points: The skin lesions were hypertrophic and eminently raised.
	4 points: The skin lesion was highly thickened, with several obvious ridges.
Scale	0 points: No scale on the surface.
	1 points: The surface of some lesions is covered with fine scales.
	2 points: The surface of most lesions is covered with flaky scales.
	3 points: The surface of most lesions is covered with non-viscous thick scales.
	4 points: All lesions were covered with viscous extremely thick scales.

### 2.6 Pathological evaluation

The skin tissue from the back of mice was fixed with 4% paraformaldehyde, embedded in paraffin, and sectioned with a thickness of 5 μm. Tissue sections were dewaxed with alcohol, stained with hematoxylin, differentiated with alcohol hydrochloride, and stained with eosin. Finally, the slices were washed and dehydrated by adding absolute ethanol. The slices were sealed, and the histopathological changes of mice were observed and photographed using a microscope. After pictures were taken, the thickness of the epidermis was measured. After photography, epidermal thickness of histopathological sections was measured by a chief of pathology using ORIGIN 8.0 software and light microscopy, and the measurement process was performed in a blind manner.

### 2.7 Immunological evaluation

Serum from mice was collected and a serum sample of 50 μL was taken from each group of mice and diluted with 1×Dilution buffer R (DAKEWE). The diluted serum samples were added to the culture plates, and antibody solution was added to detect the concentrations of Th1 and Th17 related immune molecules TNF-α, IFN-γ, IL-17A and IL-23 in mouse serum. The experimental procedure was performed according to the instructions of the ELISA kit (DAKEWE) manufacturer.

### 2.8 Data collection and statistical analysis

Statistical software SPSS13.0 was used to process the data, and the measurement data were expressed as mean ± standard deviation, and comparison between groups was performed by ANOVA analysis and linear regression analysis. The Dunnett’s test was used as *post-hoc* test of the data. P < 0.05 was considered statistically significant.

## 3 Result

### 3.1 Identification of HUCMSCs

**Figure 1:**
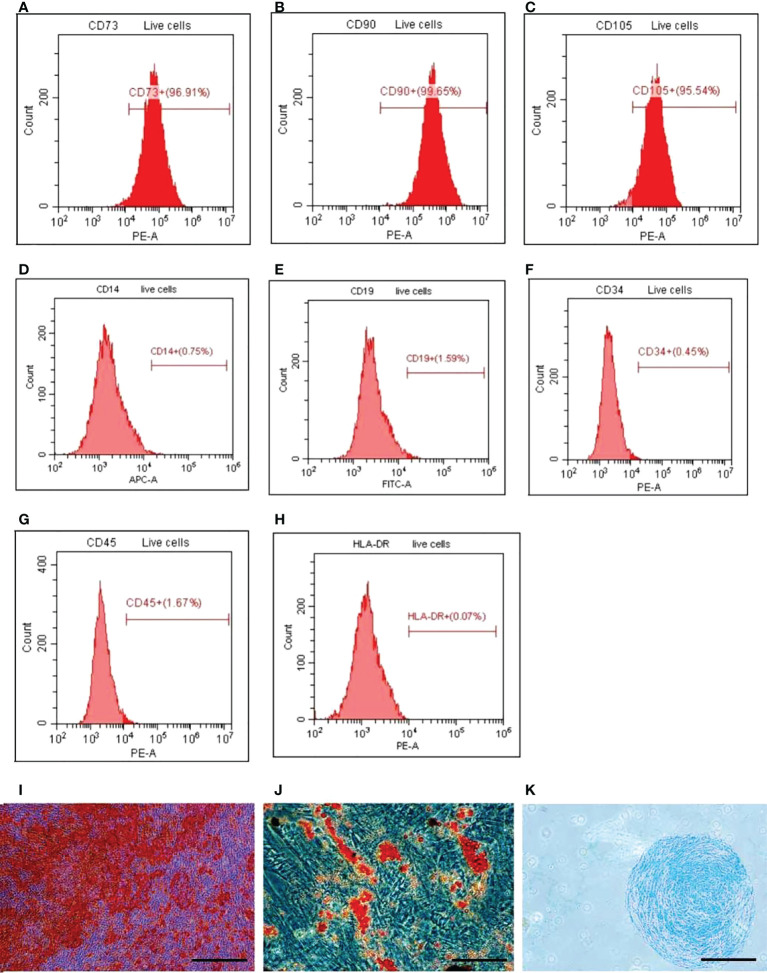
Detection of surface markers, osteogenesis, adipogenesis, and cartilage‐induced differentiation of HUCMSCs. FACS was used to detect the surface markers and ICC was used to detect the differentiation. **(A–C)** The expression of positive surface marker CD73, CD90, and CD105. **(D–H)** The expression of surface negative markers CD14, CD19, CD34, CD45, HLA‐DR. **(I)** The cells osteogenic differentiation was analyzed by alizarin red staining (scale bar = 20 μm). **(J)** The cells adipogenic differentiation was analyzed by oil red O staining (scale bar = 20 μm). **(K)** The cells chondrogenic differentiation was analyzed by alcian blue staining (scale bar = 100 μm). HUCMSCs, human umbilical cord mesenchymal stem cells.

Flow cytometry analysis confirmed that the positive rates of hUC-MSCs surface markers CD73, CD90 and CD105 were all more than 90%, while the positive rates of CD14, CD19, CD34, CD45 and HLA-DR were all less than 5% and negative (**Figures 1A–H**). The analysis of oil red O staining, alizarin red staining and alcian blue staining showed that HUC-MSCs could be successfully trans-differentiated into adipocytes, osteocytes and chondrocytes (**Figures 1I–K**). The results showed that hUC-MSCs had high purity and excellent differentiation potential.

### 3.2 Gross findings and skin lesion score results

Linear regression analysis was conducted with time as the independent variable and the scores of each part of PASI as the dependent variable. The erythema, scale, skin lesion thickness, and total score were linearly correlated with time (P < 0.001). **Table 2** shows the results. The coefficient of determination of erythema, scale, skin lesion thickness, total score, and time were 0.99, 0.99, 0.97, and 0.99, respectively. The score of skin lesions in group B continued to increase over time, and the model was successfully established.

**Table 2:** Results of correlation analysis between PASI score and time in model group B.

	Intercept	β	SE	t	p	R-squared
Erythema	0.04167	0.44881	0.02077	21.61	6.41E−07	0.9873
Scale	0	0.52143	0.02143	24.33	3.17E−07	0.99
Skin lesion thickness	0.30833	0.41548	0.02878	14.438	6.92E−06	0.972
Total score	0.325	1.30714	0.06165	21.2	7.18E−07	0.9868

SE, standard error.

**Figure 2:**
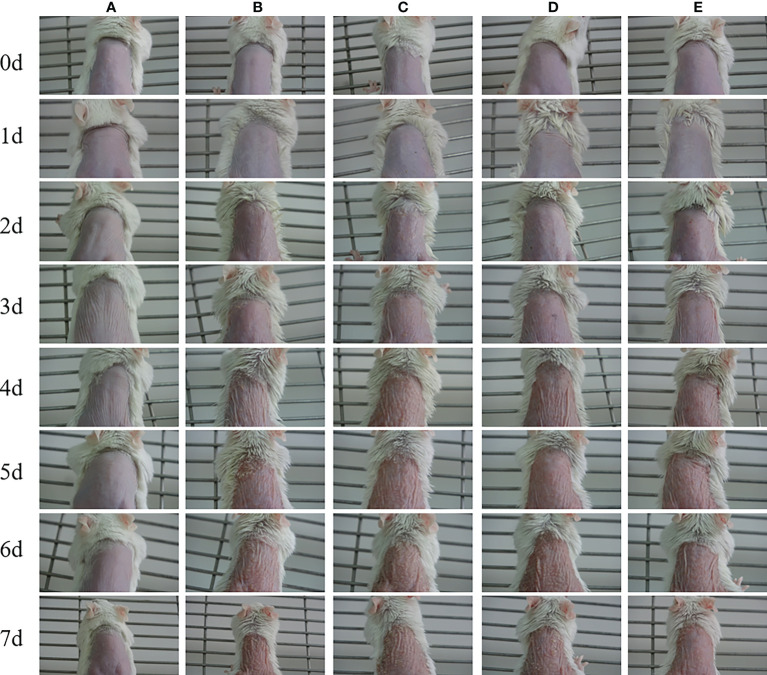
Representative images of psoriasis-like rash in each experimental group from 0 to 7 days after modeling. **(A)** Control-A; **(B)** Model-B; **(C)** hUC-MSMCs-C; **(D)** Indirubin-D; **(E)** Indirubin+hUC-MSCs-E.

Changes in skin lesions on the back of mice were observed with the naked eye (**Figure 2**). Erythema began to appear on the skin of the back of the mice 1 day after modeling, significant scale formation was observed on the third to fourth day after modeling, and the scales on the surface of the back began to increase on the fifth day after modeling. On the sixth day after modeling, the skin erythema of mice in the model group (group B) aggravated, the infiltration thickened obviously, and the surface scales continued to increase. The skin rash was similar to the appearance of human psoriasis. The psoriasis model induced by imiquimod cream +LPS/IL-12 was established.

**Figure 3:**
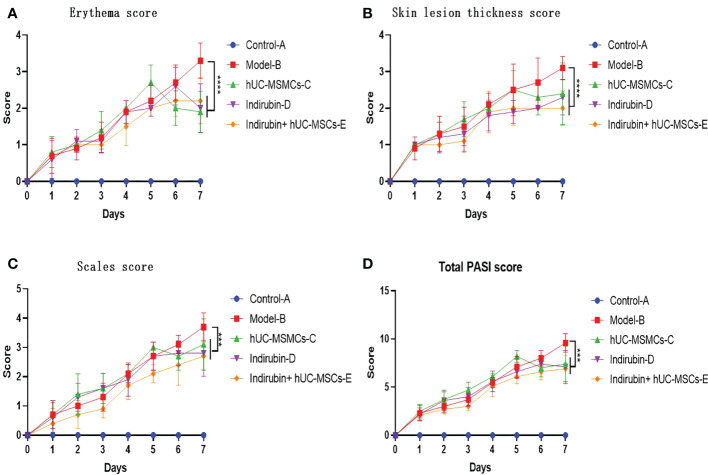
The erythema, scale, infiltration and PASI score of animals from 0 to 7 days after modeling. **(A)** Erythema score of mice in each group. **(B)** Skin lesion thickness score of mice in each group. **(C)** Scale score of mice in each group. **(D)** Total PASI score of mice in each group (one-way ANOVA/Dunnett’s test). ****P < 0.0001, ***P < 0.0001, compared with model B.

The differences in erythema scores, scale score, skin lesion thickness score, PASI score among the four groups were compared. The results of ANOVA showed that there were differences in erythema score, scale score, skin lesion thickness score and PASI score among the four groups on day 7. (P < 0.001) (**Figure 3**).

The difference of infiltration score among the four groups was compared. ANOVA results showed a difference in infiltration score among the four groups on day 7 (F = 6.842, P < 0.001) (**Table 3**). Further pairwise comparisons were performed, and Tukey’s method was used to adjust the multiplicity. The results showed significant differences between groups C and B, groups D and B, and groups E and B, and relative differences of −0.7 (P = 0.04), −0.8 (P = 0.015), and −1.1 (P < 0.001), respectively. No significant difference was found among groups D, E, and C (**Table 4**). The results showed no difference between the indirubin group and the stem cell group.

**Table 3:** Results of ANOVA of infiltration score.

	ν	SS	MS	F	P
Group	3	6.5	2.1667	6.842	0.000914
Residuals	36	11.4	0.3167		

SS, sum of square; MS, mean square.

**Table 4:** Results of pairwise comparison of infiltration score among groups.

	Relative difference	Lower confidence limits	Upper confidence limits	P
Groups C and B	−0.7	−1.3777806	−0.02221936	0.0407417
Groups D and B	−0.8	−1.4777806	−0.12221936	0.0153569
Groups E and B	−1.1	−1.7777806	−0.42221936	0.0005615
Groups D and C	−0.1	−0.7777806	0.57778064	0.9784162
Groups E and C	−0.4	−1.0777806	0.27778064	0.3972374
Groups E and D	−0.3	−0.9777806	0.37778064	0.6357029

### 3.3 Spleen index results

**Figure 4:**
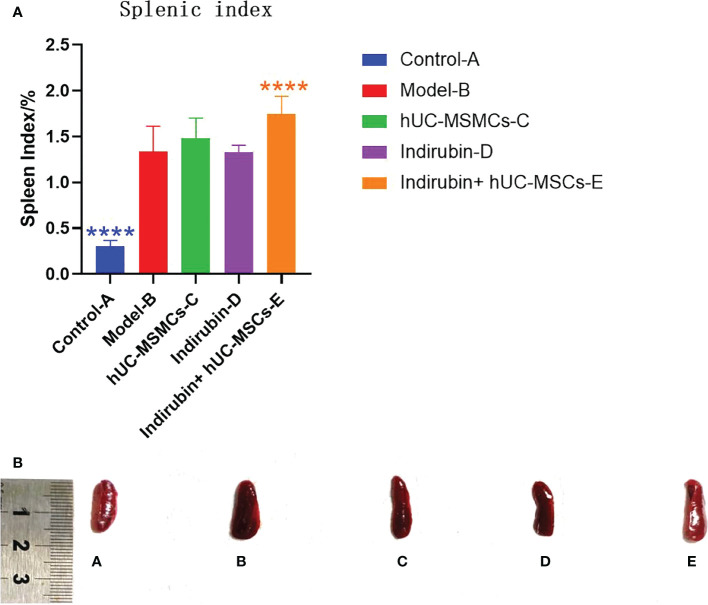
The spleen index (spleen mass/body mass) was calculated on day 7 in each group. **(A)** Spleen index of mice in each group (one-way ANOVA/Dunnett’s test). **(B)** Comparison of spleen size. ****P < 0.0001, compared with model B. A, Control-A; B, Model-B; C, hUC-MSMCs-C; D, Indirubin-D; E, Indirubin+hUC-MSCs-E.

At the end of the experiment, spleens of each group were measured, and the spleen index (spleen index = spleen mass/body mass) was calculated (**Figure 4A**). The experimental results showed that the spleen size and spleen index of each group were significantly higher than those of control group A, and statistical differences existed between model group B and the combined administration group E (P < 0.01) (**Figures 4A, B**). The mean spleen index of group A was 0.302, and that of group B was 1.338; the difference between the two groups was 1.036 (t = −11.558, P < 0.001), thus indicating significant differences. Xenogeneic exogenous stem cells in the combined administration group may cause stress response activation of the immune system of experimental animals, leading to splenomegaly, so the spleen index in the combined administration group will increase. Moreover, compared with model-B group, the spleen index of indirubin group was lower, which proved that indirubin had a certain therapeutic effect on spleen enlargement in mice.

The difference of spleen index among the four groups was compared. ANOVA results showed a difference in spleen index among the four groups on day 7 (F = 8.98, P < 0.001) (**Table 5**). Further pairwise comparisons were performed, and Tukey’s method was used to adjust the multiplicity. The results showed significant differences between groups E and B, groups C and E, and groups D and E, and the relative differences were 0.409 (P = 0.005), 0.268 (P = 0.03), and 0.42 (P < 0.001), respectively. No significant difference was found among groups C, D, and B, and between groups E and C (**Table 6**). The results showed that the combined group was superior to the stem cell group and was different from the indirubin group, and the stem cell and indirubin groups were not different from the model group.

**Table 5:** Results of ANOVA of spleen index.

	ν	SS	MS	F	P
Group	3	1.146	0.3821	8.98	0.000142
Residuals	36	1.532	0.0425		

SS, sum of square; MS, mean square.

**Table 6:** Results of pairwise comparison of spleen index among groups.

	Relative difference	Lower confidence limits	Upper confidence limits	P
Groups C and B	−0.1405282	−0.10791124	−0.38896755	0.4344881
Groups D and B	−0.0110871	−0.25952650	0.23735229	0.9993672
Groups E and B	0.4089165	0.16047707	0.65735587	0.0004679
Groups D and C	−0.1516153	−0.40005465	0.09682414	0.3678707
Groups E and C	0.2683883	0.01994892	0.51682771	0.0300334
Groups E and D	0.4200036	0.17156417	0.66844297	0.0003287

### 3.4 Pathology results

#### 3.4.1 Pathological features

**Figure 5:**
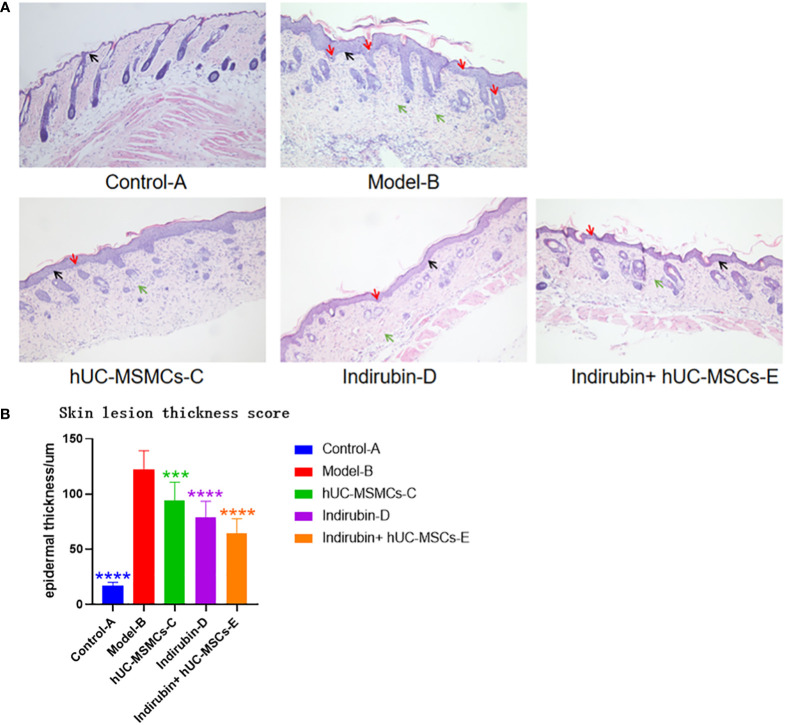
Thickness of epidermal layer was measured by H & E staining images of mice in each group. **(A)** HE staining results of pathological changes of skin tissues of mice in each group (200×). **(B)** Thickness of epidermal layer of mice in each group (one-way ANOVA/Dunnett’s test). ****P < 0.0001, ***P < 0.001, compared with model **(B)** Red arrow: Munro microabscess; Black arrow: spinous cells; Green arrow: inflammatory cells.

An observation of the dorsal skin of mice in each group (**Figure 5A**) shows that the epidermal layer of mice in control-A group is thin, and the morphology of epidermal cells is roughly normal. The epidermis of other groups was significantly thickened, being thicker than normal skin epidermis. In the model-B group, it was observed that the epidermic cells hyperkeratosis with integration parakeratosis, acanthosis cell layer thickening, epidermal sudden downward extension of in-depth dermis, and a large number of inflammatory cells were infiltrated in the dermis, which were basically the same as the pathological changes of human psoriasis. Compared with the model-B group, the hUC-MSCs-C group, indirubin-D group had significantly fewer keratinized cells, acanthosis cell layer mild hyperplasia, epidermal sudden downward extension reduced, and the inflammatory cell infiltration was significantly reduced. Compared with model-B group, the epidermal thickness of mice was reduced after drug treatment, and the epidermal thickness in hUM-MCSs combined with indirubin group was the lowest, but still higher than that of control group-A group. In indirubin+hUM-MCSs group, some mild hyperkeratinized cells were observed in the epidermis, but no inkeratinized cells were observed, the acanthosis cell layer was mildly hyperplasia. Therefore, hUM-MCSs combined with indirubin has the best effect in inhibiting skin damage and epidermal thickening of psoriasis.

#### 3.4.2 Epidermal measurement results

The thickness of the epidermal layer of the mice in each group was measured, and the results were as follows: group B (122 ± 17.0 μm) > group C (79 ± 14.3 μm) > group D (78 ± 8.7 μm) > group E (64 ± 13.1 μm) (**Figure 5B**). Statistically significant differences were found between each treatment group and model group B (P < 0.01). Compared with the other treatment groups, the combined treatment group E had the smallest epidermal thickness.

### 3.5 ELISA test results

**Figure 6:**
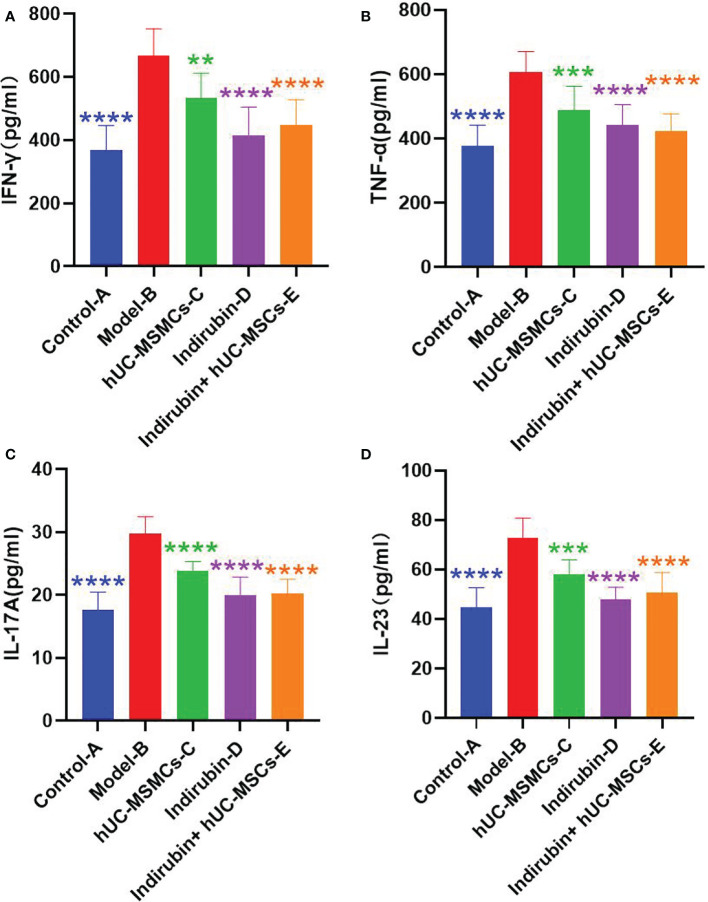
Concentrations of Th1- and Th17-related immune molecules TNF-α, IFN-γ, IL-17A, and IL-23 were detected by relative Elisa kits. **(A)** Concentrations of cytokine IFN-γ in serum of mice in each group. **(B)** Concentrations of cytokine TNF-α in serum of mice in each group. **(C)** Concentrations of cytokine IL-17A in serum of mice in each group. **(D)** Concentrations of cytokine IL-23 in serum of mice in each group (one-way ANOVA/Dunnett’s test). ****P < 0.0001, ***P < 0.001, **P < 0.01, compared with model B.

ELISA was used to detect the concentrations of Th1- and Th17-related immune molecules TNF-α, IFN-γ, IL-17A, and IL-23 in serum of mice in each group (as shown in **Tables 7**–**14** and **Figure 6**). The expression concentrations of cytokines in group B were higher than those in other groups. The concentrations of the four factors in serum of mice in each group were significantly different from those of group B (P < 0.01).

**Table 7:** Results of ANOVA of IFN-γ.

	ν	SS	MS	F	P
Group	3	376322	125441	17.82	2.95 × 10^−7^
Residuals	36	253431			

SS, sum of square; MS, mean square.

**Table 8:** Results of pairwise comparison of IFN-γ.

	Relative difference	Lower confidence limits	Upper confidence limits	P
Groups C and B	−134.82388	−235.88094	−33.76682	0.0051310
Groups D and B	−250.82234	−351.87940	−149.76528	0.0000005
Groups E and B	−218.86098	−319.91805	−117.80392	0.0000068
Groups D and C	−115.99846	−217.05552	−14.94140	0.0191699
Groups E and C	−84.03710	−185.09416	17.01996	0.1316457
Groups E and D	31.96136	−69.09571	133.01842	0.8292858

**Table 9:** Results of ANOVA of TNF-α.

	ν	SS	MS	F	P
Group	3	203527	67842	16.3	7.43 × 10^−7^
Residuals	36	149858	4163		

SS, sum of square; MS, mean square.

**Table 10:** Results of pairwise comparison of TNF-α.

	Relative difference	Lower confidence limits	Upper confidence limits	P
Groups C and B	−117.25312	−194.96316	−39.54309	0.0013716
Groups D and B	−164.29147	−242.00150	−86.58143	0.0000103
Groups E and B	−183.55267	−261.26271	−105.84264	0.0000013
Groups D and C	−47.03834	−124.74838	30.67169	0.3750147
Groups E and C	−66.29955	−144.00958	11.41048	0.1174843
Groups E and D	−19.26120	−96.97124	58.44883	0.9085997

**Table 11:** Results of ANOVA of IL-17A.

	ν	SS	MS	F	P
Group	3	621.3	207.09	37.16	4.05 × 10^−11^
Residuals	36	200.6	5.57		

SS, sum of square; MS, mean square.

**Table 12:** Results of pairwise comparison of IL-17A.

	Relative difference	Lower confidence limits	Upper confidence limits	P
Groups C and B	−5.9159186	−8.759176	−3.0726615	0.0000136
Groups D and B	−9.7532558	−12.596513	−6.9099986	0.0000000
Groups E and B	−9.5193331	−12.362590	−6.6760760	0.0000000
Groups D and C	−3.8373371	−6.680594	−0.9940800	0.0045776
Groups E and C	−3.6034145	−6.446672	−0.7601573	0.0083306
Groups E and D	0.2339227	−2.609334	3.0771798	0.9960904

**Table 13:** Results of ANOVA of IL-23.

	ν	SS	,MS	F	P
Group	3	3713	1237.6	26.57	4.05 × 10^−11^
Residuals	36	1677	46.6		

SS, sum of square; MS, mean square.

**Table 14:** Results of pairwise comparison of IL-23.

	Relative difference	Lower confidence limits	Upper confidence limits	P
Groups C and B	−14.631466	−22.851869	−6.4110625	0.0001597
Groups D and B	−24.827428	−33.047832	−16.6070250	0.0000000
Groups E and B	−22.081641	−30.302044	−13.8612373	0.0000001
Groups D and C	−10.195963	−18.416366	−1.9755593	0.0100991
Groups E and C	−7.450175	−15.670578	0.7702284	0.0873333
Groups E and D	2.745788	−5.474616	10.9661909	0.8050746

The mean IFN-γ of group A was 369.261, and the mean IFN-γ of group B was 666.478; the difference between the two groups was 17.803, which was significant (t = −8.1453, P < 0.001) (**Figure 6A**).

The difference of IFN-γ among the four groups was compared. ANOVA results showed a difference in IFN-γ among the four groups on day 7 (F = 17.82, P < 0.001) (**Table 7**). Further pairwise comparisons were performed, and Tukey’s method was used to adjust the multiplicity. The results showed significant differences between groups D and B, groups E and B, and groups D and C, and the relative differences were −134.824 (P = 0.005), 0.250.822 (P < 0.001), and −115.998 (P = 0.019), respectively. No significant difference was observed between groups E and C, and groups E and D (**Table 8**). The results showed that the indirubin group had stronger IFN-γ intervention than the stem cell group.

The mean value of TNF-α in group A was 378.823, and that in group B was 605.863; the difference between the two groups was 17.733, which was significant (t = −8.3208, P < 0.001) (**Figure 6B**).

The difference of TNF-α among the four groups was compared. ANOVA results showed a difference in TNF-α among the four groups on day 7 (F = 16.3, P < 0.001) (**Table 9**). Further pairwise comparisons were performed, and Tukey’s method was used to adjust the multiplicity. The results showed significant differences among groups C, D, E, and B, and the relative differences were −117.253 (P = 0.001), −164.291 (P < 0.001), and −183.553 (P < 0.001). No significant difference was found among groups D, E, and C (**Table 10**). No difference existed between the indirubin group and the stem cell group.

The mean value of IL-17A in group A was 17.583, and that in group B was 29.766; the difference between the two groups was 17.861, which was significant (t = −9.8441, P < 0.001) (**Figure 6C**).

The difference of IL-17A among the four groups was compared. ANOVA results showed a difference in IL-17A among the four groups on day 7 (F = 37.16, P < 0.001) (**Table 11**). Further pairwise comparisons were performed, and Tukey’s method was used to adjust the multiplicity. The results showed no significant difference between groups D and E, while significant differences existed between the other two groups (**Table 12**). No difference was observed between the indirubin group and the combined group, and the indirubin group was superior to the stem cell group.

The mean value of IL-23 in group A was 44.60, and that in group B was 72.77; the difference between the two groups was 17.996, which was significant (t = −7.833, P < 0.001) (**Figure 6D**).

The difference of IL-23 among the four groups was compared. ANOVA results showed a difference in IL-23 among the four groups on day 7 (F = 26.57, P < 0.001) (**Table 12**). Further pairwise comparisons were performed, and Tukey’s method was used to adjust the multiplicity. The results showed no significant difference among groups C, D, and E, whereas significant differences were found between the other two groups (**Table 14**). No difference existed between the indirubin group and the stem cell group and between the indirubin group and the combined group. The combined group was superior to the stem cell group.

## 4 Discussion

Psoriasis, like most autoimmune diseases, is an immune-mediated skin disease influenced by genetic and epigenetic modifications that can be triggered by environmental factors. To date, several important immune cell subsets have been found to play a role in the pathogenesis of psoriasis and other autoimmune diseases, including Th1, Th2, Treg, and Th17 cells, and the corresponding cytokines may involve IFN-γ, TNF-α, IL-23, and IL-17 (17).

hUC-MSCs are cells with self-renewal and multi-differentiation potential. They can promote tissue repair and regulate immune function by secreting a variety of cytokines. They also have low immunogenicity and have many advantages in the treatment of immune-related diseases. Reports have indicated that hUC-MSCs transplantation can treat psoriasis patients (4). hUC-MSCs are a safer psoriasis treatment option than topical glucocorticoids and can effectively avoid the adverse reactions caused by topical glucocorticoids on the epidermis. Electron microscopy showed that the epidermis of psoriasis cured by hUC-MSCs had uniform thickness, complete epidermis layers, clear boundary between the dermis and the epidermis, complete basement membrane, and no gap between tightly connected basal layer cells and basement membrane. Moreover, hUC-MSCs treatment could also reduce the expression of CD4, CD8 and CD31 in T cells (18). hUC-MSCs can significantly reduce the severity and development of psoriasis, inhibit the infiltration of immune cells into the skin, and downregulate the expression of various proinflammatory cytokines and chemokines, including first inhibiting neutrophil function and then downregulating the production of type I interferon in plasmacytoid dendritic cells (19). In this study, hUC-MSCs were used to treat imiquimod-induced BALB/c psoriasis mouse model. hUC-MSCs could significantly reduce the skin lesion score of the psoriasis mouse model and also reduce the epidermal thickness of skin lesions. In addition, Th1 cytokines (TNF-α and IFN-γ) and IL-27 and Th17 cytokines (IL-17A and IL-23) in serum of mice could be significantly inhibited by hUC-MSCs. Compared with the model group, hUC-MSCs in this experiment did not significantly reduce the spleen index of mice (20), which may be due to the different experimental protocols. The difference is that the spleen index of mice was directly proportional to the concentration of UC-MSCs in previous studies (6), this may be because xenogeneic exogenous stem cells introduced into mice may activate the immune system stress response in mice, leading to spleen enlargement, and then increase the spleen index of mice. Therefore, UC-MSCs in the combined administration group may lead to the increase of spleen index in mice. Compared with the model group, the spleen index in the indirubin group showed a downward trend, which proved that indirubin could improve the prognosis of mice. Considering that the mechanism of UC-MSCs in the treatment of psoriasis is different from immunosuppression, spleen index cannot be simply used as a measurement index, which will be further discussed in subsequent research.

Indirubin is one of the main active components in indigo naturalis. Current studies have shown that indirubin has a dose-dependent therapeutic effect on alleviating psoriasis-like skin lesions in mice (21), among which CD274 is a regulatory factor of indirubin-mediated effect on psoriasis-like skin lesions in mice (14). Indirubin attenuates imiquimod-induced psoriatic dermatitis mainly by reducing the inflammatory response mediated by γδT cells generated by IL-17A involving Jak3/Stat3 activation (22). Therefore, indirubin and its preparations may be developed as new drugs for the treatment of psoriasis. An *in vitro* experiment found that indirubin could significantly reduce the mRNA expression levels of proinflammatory cytokines IL-1β, IL-6, and TNF-α in HaCaT cells and cell culture supernatant, and the differences were statistically significant (P < 0.05). Moreover, the inhibitory effect was enhanced with the increase in the concentration of indirubin. A certain concentration dependence is observed (23). According to relevant literature analysis, the scholar treated mice with 50 mg/kg indirubin instillation to explore the therapeutic effect of indirubin on CD274 and skin lesions. The experimental results were clear and the mice had normal activity (22). Therefore, mice treated with 50 mg/kg indirubin in this experiment showed normal activity without obvious toxic and side effects, and the experimental results were significant.

This study confirmed that the three groups had significant inhibitory effects on IFN-γ, IL-17A, and IL-23 in mouse serum. However, further intergroup comparison indicated that the indirubin group had more obvious effects on the above three indicators than the hUC-MSCs group did. These results suggest that indirubin has a strong inhibitory effect on the psoriasis-related IL-23/Th17/IL-17 axis and Th1/IFN-γ axis. In addition, although the improvement of PASI score was significantly different among the three groups, the indirubin group was significantly better than the hUC-MSCs group in reducing scale, considering that indirubin can inhibit the mitosis of cells in psoriatic lesions and regulate cell keratinization. The systematic use of UC-MSCs therapy combined with indirubin external preparation may further improve the efficacy.

After the combination of hUC-MSCs and indirubin, the epidermis was the thinnest in the pathological sections of mice, thereby suggesting that the combination of hUC-MSCs and indirubin could exert a synergistic effect to better inhibit the proliferation of psoriasis epidermis. The spleen index of mice increased significantly after the combination of drugs, but single treatment of indirubin or hUC-MSCs had no obvious effect on the spleen index of mice, which may be due to the synergistic effect of the two groups of drugs in strengthening the regulation of immune function.

In general, according to the PASI score and pathological analysis of skin lesions, this study confirmed that hUC-MSCs, traditional Chinese medicine indirubin, and the combination of the above two groups had therapeutic and intervention effects on skin lesions of mice with psoriasis. Moreover, the concentrations of IFN-γ, TNF-α, IL-17A, and IL-23 in skin lesions and serum of mice decreased significantly, suggesting that hUC-MSCs and indirubin are involved in the process of immune regulation in the treatment of psoriasis, and the targets and focuses are different. This subject needs to be further explored in future research.

## Data availability statement

The original contributions presented in the study are included in the article/supplementary materials. Further inquiries can be directed to the corresponding authors.

## Ethics statement

Experiments were performed under a project license (No. 2016LL003) granted by institutional board of The Fifth People’s Hospital of Hainan Province, in compliance with The Fifth People’s Hospital of Hainan Province guidelines for the care and use of animals.

## Author contributions

(I) Conception and design: JY; (II) Administrative support: HW; (III) Provision of study materials or patients: HW, YL, and XL; (IV) Collection and assembly of data: XL; (V) Data analysis and interpretation: All authors; (VI) Manuscript writing: JY; (VII) Final approval of manuscript: All authors.

## Funding

This work was supported by Hainan Province Clinical Medical Center and the funding project “Effects of indigo naturalis and Arnebia on differentiation and barrier function of epidermal stem cells in psoriasis response research (Hainan Natural Science Foundation, 20158342)”

## Conflict of interest

Authors HW, HW, FX, CJ, DS, and HZ were employed by Asia Stem Cell Regenerative Pharmaceutical Co. Ltd.

The remaining authors declare that the research was conducted in the absence of any commercial or financial relationships that could be construed as a potential conflict of interest.

## Publisher’s note

All claims expressed in this article are solely those of the authors and do not necessarily represent those of their affiliated organizations, or those of the publisher, the editors and the reviewers. Any product that may be evaluated in this article, or claim that may be made by its manufacturer, is not guaranteed or endorsed by the publisher.
